# The hematopoietic regulator TAL1 is required for chromatin looping between the β-globin LCR and human γ-globin genes to activate transcription

**DOI:** 10.1093/nar/gku072

**Published:** 2014-01-25

**Authors:** Won Ju Yun, Yea Woon Kim, Yujin Kang, Jungbae Lee, Ann Dean, AeRi Kim

**Affiliations:** ^1^Department of Molecular Biology, College of Natural Sciences, Pusan National University, Pusan 609-735, Korea and ^2^Laboratory of Cellular and Developmental Biology, NIDDK, NIH, Bethesda, MD 20892, USA

## Abstract

TAL1 is a key hematopoietic transcription factor that binds to regulatory regions of a large cohort of erythroid genes as part of a complex with GATA-1, LMO2 and Ldb1. The complex mediates long-range interaction between the β-globin locus control region (LCR) and active globin genes, and although TAL1 is one of the two DNA-binding complex members, its role is unclear. To explore the role of TAL1 in transcription activation of the human γ-globin genes, we reduced the expression of TAL1 in erythroid K562 cells using lentiviral short hairpin RNA, compromising its association in the β-globin locus. In the TAL1 knockdown cells, the γ-globin transcription was reduced to 35% and chromatin looping of the ^G^γ-globin gene with the LCR was disrupted with decreased occupancy of the complex member Ldb1 and LMO2 in the locus. However, GATA-1 binding, DNase I hypersensitive site formation and several histone modifications were largely maintained across the β-globin locus. In addition, overexpression of TAL1 increased the γ-globin transcription and increased interaction frequency between the ^G^γ-globin gene and LCR. These results indicate that TAL1 plays a critical role in chromatin loop formation between the γ-globin genes and LCR, which is a critical step for the transcription of the γ-globin genes.

## INTRODUCTION

The β-globin locus adopts a specific chromatin structure in erythroid cells where the globin genes are highly transcribed. DNase I hypersensitive sites (HSs) are formed in the upstream locus control region (LCR), and histone modifications associated with active chromatin, such as H3 acetylation and H3K4 methylation, are established in the LCR and transcribed globin genes (1). In addition, HSs of the LCR are juxtaposed to the transcribed globin genes, generating chromatin loops (2,3). The formation of the active β-globin locus structure requires erythroid-specific transcription activators that bind to the LCR HSs and gene promoters and associate with co-activators to modify chromatin structure.

GATA-1 and NF-E2 are erythroid factors with central roles in erythropoiesis and specific roles in transcription activation of the β-like globin genes. GATA-1 plays a role in acetylating histones in the β-globin locus by recruiting histone acetyltransferase CBP into the LCR HSs (4–7). NF-E2 contributes to HS formation in the LCR and recruits Brg1, the ATPase component of SWI/SNF nucleosome remodeling complex (6,8). The proximity between the LCR and mouse β-globin gene requires GATA-1 as shown in murine G1E cells (9). GATA-1 and NF-E2 are both required for chromatin loop formation between the LCR HSs and active ^G^γ-globin gene in human erythroid K562 cells (6), although a study using knockout mice shows that NF-E2 is dispensable for LCR/β-globin loop formation in this context, likely because of the availability of compensatory factors (10).

SCL/TAL1 (hereafter TAL1) is a basic helix-loop-helix protein that is essential for the development of all hematopoietic lineages (11,12). It is required for erythropoiesis in mice (13), and its enforced expression promotes the erythroid differentiation of progenitor cells (14–16). Globin gene transcription fails to be induced in differentiated TAL1 null embryonic stem cells (17). TAL1 functions as a heterodimer with E protein that, together with GATA-1, is the DNA-binding component of a pentameric complex including the erythroid LIM-only protein, LMO2, and the more widely expressed protein, NLI/Ldb1 (hereafter Ldb1) (18). Genome-based studies indicate that the complex occupies a composite E box-GATA motif that is common in promoters and regulatory regions of erythroid genes (19–23). NF-E2 binding motifs are distinct from these sites.

Previous studies have shown that GATA-1 is required for chromatin loop formation in the β-globin locus, as is Ldb1 (6,9,24), and it seems likely that GATA-1 carries out this function as a component of the Ldb1 complex (23). However, whether TAL1 is required for long-range activation of globin genes is unclear. Moreover, it is unknown whether GATA-1 and TAL1, the two DNA-binding members of the Ldb1 complex, have overlapping or distinct roles in Ldb1 complex formation and chromatin looping in the β-globin locus. Here, we have studied the role of TAL1 in these activities by reducing or increasing its expression in human erythroid K562 cells where the γ-globin genes are transcribed. The results show that TAL1 is required for LCR/γ-globin looping in the human β-globin locus and indicate distinctive roles for TAL1 and GATA-1. Furthermore, the results indicate the importance of chromatin loop formation for γ-globin gene transcription.

## MATERIALS AND METHODS

### TAL1 knockdown and overexpression using lentiviral vectors in K562 cells

TAL1-directed TRC lentiviral short hairpin RNA (shRNA) vectors and control shRNA vector (pLKO.1) were purchased from Sigma. Lentiviruses were produced by transfecting shRNA vectors and Virapower packing mix (Invitrogen) into 293FT cells according to the manufacturer’s instructions (6). Transduction of viral particles into K562 cells was performed in the presence of 6 µg/ml of polybrene, and 2 µg/ml of puromycin was added at 72 h after the transduction. Cells were grown for one to two weeks in RPMI 1640 medium containing 10% FBS. Knockdown of TAL1 protein was confirmed by western blot analysis.

TAL1 complementary DNA (cDNA) was cloned into the pLenti6.3/V5-DEST expression vector (Invitrogen). TAL1 expression vector was transfected into 293FT cells with Virapower packing mix to produce lentiviruses. Transduction of viral particles into K562 cells was performed in the presence of 6 µg/ml of polybrene, and 5 µg/ml of blasticidine was added after 72 h. Overexpression of TAL1 was confirmed by western blot analysis. The pLenti6.3/V5-GW/lacZ was used as a control vector to generate control cells.

### Reverse transcription-PCR

RNA was prepared from 2 × 10^6^ cells using the RNeasy Plus Mini Kit (Qiagen) and reverse transcribed with random hexamers using the Superscript III first-strand synthesis system as suggested by the manufacturer (Invitrogen). Specific cDNA for the β-globin locus and transcriptional activators, TAL1, GATA-1 and NF-E2, was amplified by quantitative polymerase chain reaction (PCR) using TaqMan chemistry and SYBR green fluorescence, respectively. In PCR using TaqMan probes, the relative intensity of specific cDNA sequences in the β-globin locus was compared with a genomic DNA standard using the comparative Ct method and then normalized with the relative intensity for the actin gene. In PCR using SYBR green fluorescence, the relative intensity was determined by comparing specific cDNA with the actin cDNA. 

### Quantitative PCR

**Figure 1.**
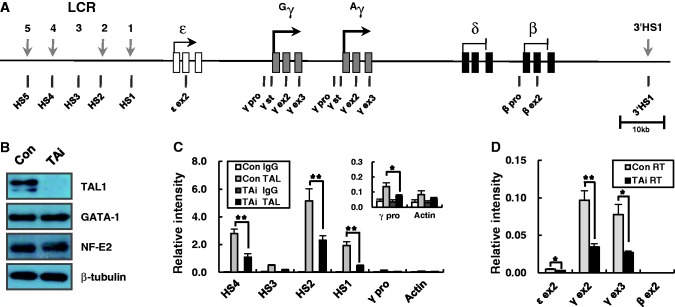
Knockdown of TAL1 in erythroid K562 cells. (**A**) The human β-globin locus is presented. Vertical black arrows indicate DNase I hypersensitive sites (HSs) in the LCR. The exons of the globin genes are represented by squares. Vertical bars named below the diagram denote the locations of TaqMan amplicons used in quantitative PCR. (**B**) TAL1, GATA-1 and p45/NF-E2 were detected by western blotting in protein extract from K562 cells expressing a control or TAL1 shRNA. Blotting with β-tubulin antibody was used as experimental control. (**C**) ChIP was performed with TAL1 antibodies in control and TAL1 knockdown K562 cells. Relative intensity was determined by quantitatively comparing input with immunoprecipitated DNA for the indicated amplicons. The data for the γ-promoter region are presented in inset graph with different Y scale. Actin served as negative control. Normal goat IgG (IgG) served as experimental control. (**D**) Transcripts of the globin genes were measured in the K562 cells by quantitative RT-PCR using primers and TaqMan probes for exons of the ε-, γ- and β-globin genes. Relative intensity of specific cDNA sequences was determined by comparing with a genomic DNA standard and then normalizing to the intensity of the actin gene. The results of four to five independent experiments ± SEM are graphed. *P < 0.05, **P < 0.01..

DNA obtained from all experiments was quantitatively analyzed using 7300 Real-time PCR system (Applied Biosystems). PCR was carried out with 200 nmol of TaqMan probes and 900 nmol of primers in a 10 μl of reaction volume for cDNA and DNA obtained from chromatin immunoprecipitation (ChIP) and MNase digestion. DNA obtained from 3 C experiment was amplified using SYBR green fluorescence in a 10 μl of reaction volume. Data were collected at the threshold where amplification was linear. The locations of amplicon containing TaqMan probe in the β-globin locus are indicated in Figure 1A. Sequences of primers, TaqMan probes and primers for 3C are provided in our previous study (6).

### Chromatin immunoprecipitation

ChIP was carried out as described (25). Briefly, K562 cells (2 × 10^7^) were cross-linked with 1% formaldehyde and then nuclei were isolated by cell lysis. MNase digestion and sonication were performed with nuclei to produce chromatin at a mononucleosome size. Fragmented chromatin was incubated with antibodies after pre-clearing. Protein–DNA complexes were recovered with protein A or G agarose beads, and DNA was purified after reverse cross-linking.

Antibodies used in this study were purchased from Santa Cruz Biotechnology for TAL1 (sc-12984), GATA-1 (sc-1233), p45/NF-E2 (sc-22827), Brg1 (sc-10768), CBP (sc-369), Pol II (sc-899) and Ldb1 (sc-11198), from Millipore for H3ac (06-599), H3K4me2 (07-030) and H3K27me2 (07-452), from Abcam for H3K27ac (ab4729), H3K36me3 (ab9050), Rad21 (ab992) and SMC3 (ab9263) and from R&D systems for LMO2 (AF2726). Normal rabbit IgG (sc-2027) and goat IgG (sc-2028) were purchased from Santa Cruz Biotechnology. 

### MNase sensitivity assay

Nuclei were prepared from K562 cells (2 × 10^7^) as described previously (26,27). Aliquots of 3 × 10^6^ nuclei were digested with 20–160 U micrococcal nuclease (MNase) in a 100 μl volume for 5 min at 25°C. DNA was purified and run on a 1.2% agarose gel to visualize the level of digestion. The digested DNA was quantitatively compared with undigested DNA by PCR. Sensitivity was determined by normalizing the difference of amount between digested DNA and undigested DNA in each primer pairs to the difference in the actin gene at each MNase concentration.

### Chromosome conformation capture

Chromosome conformation capture (3C) assay was performed as described with reduced number of cells (6,28). K562 cells were cross-linked with 1% formaldehyde, and nuclei were prepared from ∼1–2 × 10^6^ cells. Eight hundred units of Hind III restriction enzyme were used to digest DNA for overnight, and the digested DNA was ligated with T4 ligase. The ligated DNA was purified after reverse cross-linking. The 3C products were quantitatively amplified by PCR using SYBR green as fluorescence dye. To correct for the differences of ligation efficiency between fragments and the difference of PCR efficiency between primer sets, control templates were prepared by mixing, digesting and ligating equimolar amounts of the PCR fragments spanning the restriction enzyme sites and same amount of genomic DNA (28). The ligation between two fragments was analyzed using the reverse direction primer of each fragment except one as shown in Figure 4A. The relative cross-linking frequency between two fragments was determined by comparing DNA ligated in 3C samples with DNA ligated randomly in control templates and then by normalizing with the cross-linking frequency in the Ercc3 gene (29).

## RESULTS

### TAL1 is required for transcription of the γ-globin genes in erythroid K562 cells

TAL1 binds to the LCR HSs in human β-globin loci transcribing the γ- or β-globin genes (22,24,30,31) and knockdown of TAL1-reduced β-globin transcription in differentiated human hematopoietic progenitors (22). To explore the role of TAL1 in the transcription of the γ-globin genes, we inhibited its expression using shRNA in erythroid K562 cells. TAL1 protein was reduced in the knockdown cells as shown by western blot, but GATA-1 and NF-E2 protein levels were unaffected by the knockdown (Figure 1B). ChIP assay showed that TAL1 binding was strongly reduced at the LCR HS4, HS2 and HS1 sites in the knockdown cells compared with control cells (Figure 1C). At the γ-globin promoter, TAL1 was detected, even if the level is low, and reduced by the knockdown. TAL1 binding at the γ-globin promoter is not strong compared with the LCR HSs even in CD34+ human erythroid progenitor cells that express high levels of γ-globin, levels >50 fold higher than those seen in K562 cells (30). The γ-globin transcripts were decreased to 35% in the TAL1 knockdown cells compared with control cells as measured by quantitative reverse transcription-PCR (RT-PCR) (Figure 1D). These results support the idea that TAL1 occupancy in the human β-globin locus is required for transcription of the γ-globin genes.

### GATA-1 and NF-E2 occupancy in the β-globin LCR HSs is insensitive to TAL1 reduction

**Figure 2.**
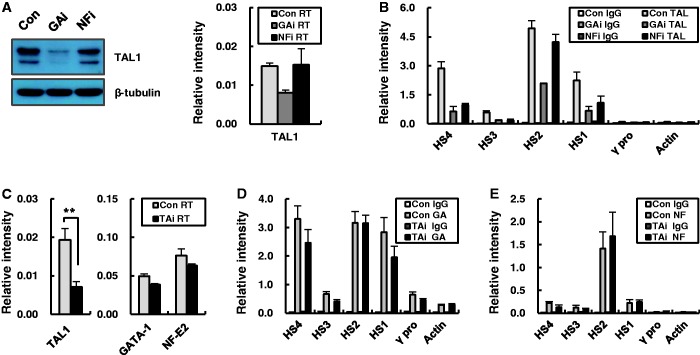
Bindings of GATA-1 and NF-E2 at the β-globin LCR HSs in TAL1 knockdown K562 cells. (**A**) Expression of TAL1 was analyzed by western blot (left panel) and quantitative RT-PCR (right panel) in K562 cells expressing a control, GATA-1 or NF-E2 shRNA. (**B**) TAL1 binding at the β-globin LCR HSs was analyzed in these cells by ChIP. (**C**) Transcripts of the TAL1, GATA-1 and NF-E2 genes were measured in K562 cells expressing a control or TAL1 shRNA by quantitative RT-PCR. GATA-1 binding (**D**) and NF-E2 binding (**E**) were analyzed in the control and TAL1 knockdown K562 cells by ChIP. Relative intensity in ChIP assay was determined as described in Figure 1. Actin served as negative control. Normal goat or rabbit IgG (IgG) served as experimental control. The results of two to four independent experiments ± SEM are graphed. **P < 0.01.

In our previous study, GATA-1 knockdown in K562 cells reduced expression of NF-E2, although the expression of GATA-1 and its occupancy at HSs sites in the β-globin LCR were largely maintained in NF-E2 knockdown cells (6). We examined the effects of GATA-1 or NF-E2 knockdown on TAL1 in K562 cells. TAL1 protein and messenger RNA (mRNA) levels and occupancy at the β-globin LCR HSs were reduced by GATA-1 knockdown (Figure 2A and B). However, TAL1 expression was maintained in NF-E2 knockdown cells and its occupancy was largely unaffected at LCR HS2, although it was reduced at the other HSs. These data and our previous observations (6) are consistent with studies in murine erythroid cells showing that *Nfe2* and *Tal1* are targets of GATA-1 (32,33). Next, we examined the effect of TAL1 knockdown on GATA-1 and NF-E2. TAL1 mRNA was reduced to ∼40% of the level in control cells by the knockdown (Figure 2C). However, TAL1 knockdown did not impact mRNA levels of GATA-1 or NF-E2 and occupancy of these factors in the LCR HSs was largely maintained, although small increases or decreases were detected at some HSs (Figure 2D and E). The lack of response of GATA-1 and NF-E2 to TAL1 reduction is similar to what has been observed in human erythroid progenitor cells (22). These results indicate that TAL1 expression and β-globin locus occupancy are dependent on GATA-1 but GATA-1 and NF-E2 expression and their occupancy in the β-globin locus are largely independent of TAL1 in K562 cells.

### HSs formation and histone modifications in the β-globin locus are not affected by TAL1 knockdown in K562 cells

**Figure 3.**
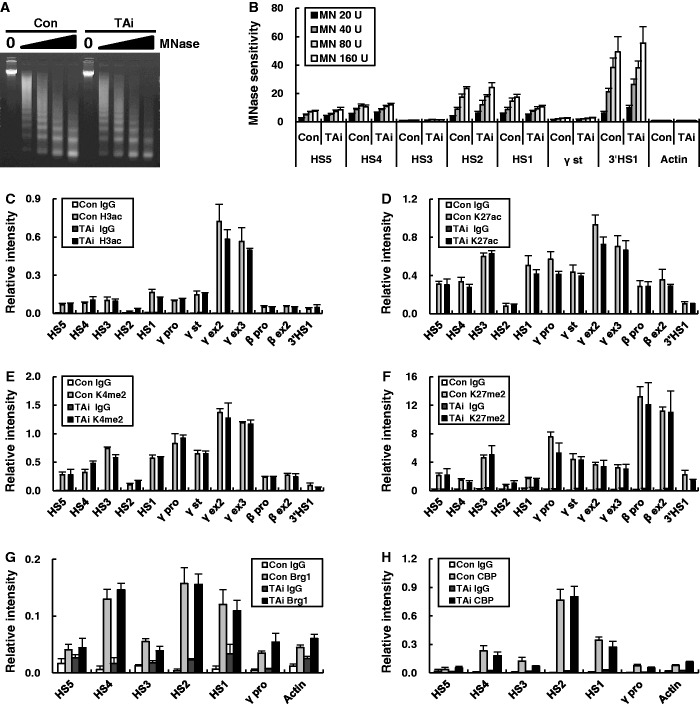
MNase sensitivity and histone modifications of the β-globin locus in TAL1 knockdown K562 cells. (**A**) Nuclei of K562 cells expressing a control or TAL1 shRNA were digested with 20–160 U MNase. DNA extracted from the digest was run on 1.2% agarose gel. (**B**) MNase sensitivity was determined by quantitatively comparing digested DNA with undigested DNA and normalizing to the sensitivity of the actin gene at each concentration. The results are averages of four nuclei preparations ± SEM. Histone modifications were analyzed by ChIP with antibodies specific to H3K9/K14ac (**C**), H3K27ac (**D**), H3K4me2 (**E**) and H3K27me2 (**F**) in K562 cells expressing each shRNA. Relative intensity was determined by quantitatively comparing immunoprecipitated DNA with input for the indicated amplicons and then by normalizing to the intensity of the actin gene. Association of Brg1 (G) and CBP (H) was analyzed by ChIP as described in Figure 1. Normal rabbit IgG (IgG) served as experimental control. The results of two to four independent experiments ± SEM are graphed.

In the β-globin locus transcribing the γ-globin genes, GATA-1 and NF-E2 play distinctive roles in HSs formation and locus histone modification (6). To ask whether TAL1 has a role in these processes independent from GATA-1 and NF-E2, sensitivity to MNase and several histone modifications was analyzed in TAL1 knockdown K562 cells. MNase cleaves HSs yielding a similar pattern to DNase I (34). Surprisingly, MNase sensitivity at the LCR HSs and boundary HSs was unchanged by TAL1 knockdown (Figure 3A and B). Likewise, the locus-wide patterns of active epigenetic marks, including acetylation of histone H3K9/14 and H3K27 and dimethylation of histone H3K4 were similar in TAL1 knockdown cells compared with control cells as was the pattern of the H3K27 repressive mark, which appears over the non-expressed adult β-globin gene (Figure 3C–F). In agreement with these results, enrichment of Brg1, the catalytic subunit of the SWI/SNF nucleosome remodeling complex and the histone acetyltransferase CBP at the LCR was unaffected by TAL1 knockdown (Figure 3G and H). However, histone H3K36me3, which is dependent on RNA polymerase elongation was reduced in the exons of the γ-globin genes in parallel with reduced RNA polymerase II occupancy in TAL1 knockdown cells (Supplementary Figure S1). Together, these results show that TAL1 does not have a role in HSs formation and histone modifications, including H3K9/14ac, H3K27ac and H3K4me2 in the β-globin locus in K562 cells. These chromatin modifications appear to depend on GATA-1 and NF-E2 (6).

### Proximity between the ^G^γ -globin gene and LCR HSs requires TAL1

**Figure 4.**
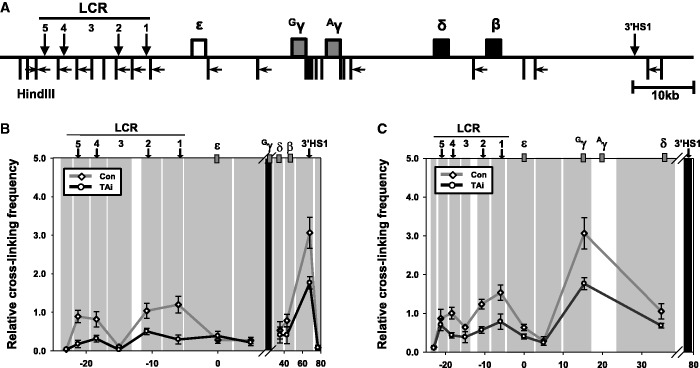
Relative proximity between HSs and the ^G^γ-globin gene in the β-globin locus in the TAL1 knockdown K562 cells. The 3C assay was performed with Hind III restriction enzyme in K562 cells expressing a control or TAL1 shRNA. (**A**) Hind III sites and PCR primers in the β-globin locus were represented by vertical bars and horizontal arrows, respectively. The black shading represents the anchor fragment for ^G^γ-globin gene (**B**) and 3′HS1 (**C**) in PCR. The gray shadings are fragments generated by Hind III digestion. Relative cross-linking frequency was determined by quantitatively comparing ligated DNA in cross-linked chromatin with control DNA and then by normalizing to the cross-linking frequency in the Ercc3 gene. The results are averages of four to five independent experiments ± SEM.

Close positioning between the human ^G^γ-globin gene and LCR is observed when the gene is active and is disrupted by the reduction of GATA-1 or NF-E2 in K562 cells (6,35). To address the role of TAL1 in chromatin loop formation, the 3C assay was performed in control cells and TAL1 knockdown K562 cells (Figure 4A). Hind III cleavage was used to obtain a high resolution view of the long-range interactions of individual LCR HSs. Using the ^G^γ-globin gene as a viewpoint, we observed that the cross-linking frequency between fragment containing the gene and fragments containing LCR HSs was decreased in the TAL1 knockdown cells (Figure 4B). Proximity of the gene with locus-flanking CTCF insulators, HS5 and 3′HS1, was also reduced by TAL1 knockdown, indicating the requirement of TAL1 for the active chromatin hub wherein the β-globin LCR HSs and the active genes are juxtaposed with HS5 and 3′HS1 (28). No significant effect of TAL1 knockdown on 3′HS1/HS5 interaction was observed when 3′HS1 was used as the viewpoint (Figure 4C). Thus, in addition to GATA-1, TAL1 appears to play a role in chromatin looping between the ^G^γ-globin gene and LCR, whereas HS5 and 3′HS1 CTCF insulator sites appear to interact with each other independent of TAL1.

### Ldb1 and LMO2 occupancy in the LCR HSs are reduced by TAL1 knockdown

**Figure 5.**
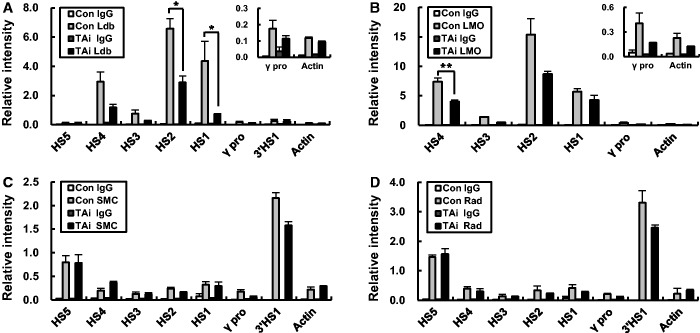
Binding of cofactors in the human β-globin locus in the TAL1 knockdown K562 cells. ChIP was performed with Ldb1 (**A**), LMO2 (**B**), SMC3 (**C**) and Rad21 (**D**) antibodies in K562 cells expressing a control or TAL1 shRNA. Relative intensity in ChIP assay was determined as described in Figure 1. Actin served as a control. Normal goat or rabbit IgG (IgG) served as experimental control. The results of two or three independent experiments ± SEM are graphed. *P < 0.05.

Next, to further explore the role of TAL1 in chromatin loop formation, the occupancy of the loop-mediating Ldb1 and LMO2 proteins was examined in the β-globin locus. Ldb1 and LMO2 were less occupied at the LCR HSs in TAL1 knockdown cells (Figure 5A and B). Their occupancies were slightly reduced at the γ-globin promoter. However, SMC3 and Rad21, subunits of the cohesin complex known to occupy HS5 and 3′HS1, bind to these sites at similar levels in the knockdown cells and control cells (Figure 5C and D). These results indicate that TAL1 plays a role in chromatin loop formation by recruiting or stabilizing the Ldb1 complex. Both TAL1 and GATA-1 interact with the bridging protein LMO2 (18,36,37). In addition, the results suggest that GATA-1 binding alone to E box–GATA-1 sites might not be enough to recruit the complex into the LCR HSs.

### Overexpression of TAL1 increases the frequency of interaction between the active ^G^γ-globin gene and regulatory elements in the β-globin locus

**Figure 6.**
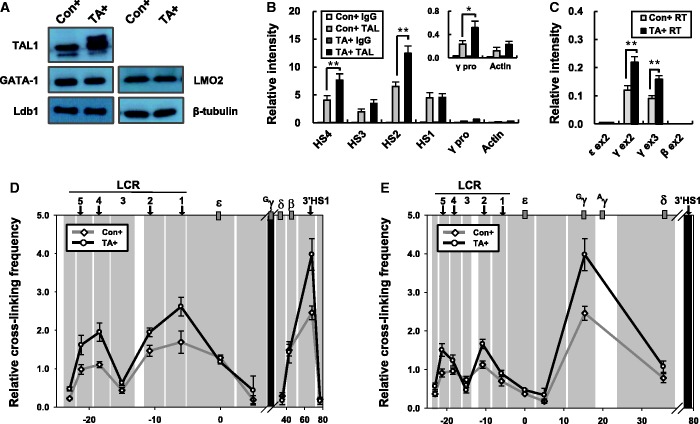
Relative proximity between HSs and the ^G^γ-globin gene in the β-globin locus in the TAL1 overexpressing K562 cells. (**A**) TAL1, GATA-1, Ldb1 and LMO2 were detected by western blotting in protein extract from K562 cells expressing a control or TAL1 cDNA. Blotting with β-tubulin antibody was used as experimental control. (**B**) ChIP was performed with TAL1 antibodies in control or TAL1 overexpressing K562 cells. Relative intensity was determined as described in Figure 1. Actin served as negative control. Normal goat IgG (IgG) served as experimental control. The results are averages of five independent experiments ± SEM. (**C**) Transcripts of the globin genes were measured in the K562 cells as described in Figure 1. The results of six independent experiments ± SEM are graphed. The 3C assay was performed with Hind III restriction enzyme in the K562 cells. Fragment for ^G^γ-globin gene (**D**) or 3′HS1 (**E**) was used as an anchor in PCR. Relative cross-linking frequency was determined as described in Figure 4. The results are averages of four to five independent experiments ± SEM. *P < 0.05, **P < 0.01.

Reduced TAL1 occupancy diminished chromatin loop formation between the ^G^γ-globin gene and LCR. To further examine the role of TAL1 in chromatin loop formation, we overexpressed TAL1 in K562 cells using a lentiviral vector. An increase of TAL1 protein was observed in the overexpressing cells, whereas protein levels of GATA-1, Ldb1 and LMO2 were unaffected (Figure 6A). TAL1 overexpression increased its occupancy at LCR HS4 and HS2 and γ-globin promoter (Figure 6B), and transcription of the γ-globin gene was increased 1.8-fold (Figure 6C). In the 3C assay, using the ^G^γ-globin gene as the viewpoint, TAL1 overexpression increased cross-linking frequency to the LCR HSs and 3′HS1 (Figure 6D). The cross-linking frequency between HS5 and 3′HS1 was also increased when 3′HS1 was used as the viewpoint (Figure 6E). These 3C results show that the LCR HS5-1, the ^G^γ-globin gene and 3′HS1 interact more frequently in TAL1 overexpressing cells. Together with the data in Figure 4, these results show that TAL1 plays an important role in chromatin loop formation in the human β-globin locus. More frequent loop contacts resulting from TAL1 overexpression appear to contribute to the increase of the γ-globin transcription.

### Ldb1 and LMO2 occupancy in the LCR is increased by TAL1 overexpression but GATA-1 occupancy is not

**Figure 7.**
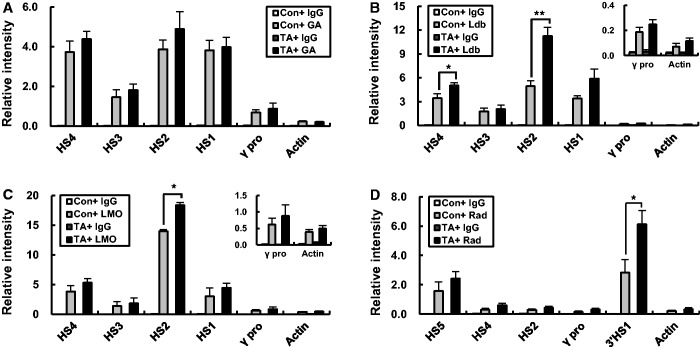
Binding of transcriptional activators and cofactors in the human β-globin locus in the TAL1 overexpressing K562 cells. ChIP was performed with GATA-1 (**A**), Ldb1 (**B**), LMO2 (**C**) and Rad21 (**D**) antibodies in K562 cells expressing a control or TAL1 cDNA. Relative intensity in ChIP assay was determined as described in Figure 1. Actin served as a control. Normal goat or rabbit IgG (IgG) served as experimental control. The results of three or four independent experiments ± SEM are graphed. *P < 0.05, **P < 0.01.

To ask whether increased TAL1 expression and LCR occupancy affect the binding of other proteins in the β-globin locus, the occupancy of GATA-1, Ldb1, LMO2 and Rad21 was analyzed at the LCR HSs and γ-globin promoter. Occupancy of GATA-1 was not changed in TAL1 overexpressing cells (Figure 7A). In contrast, Ldb1 and LMO2 were more highly detected at HS2 when TAL1 was overexpressed, and their binding patterns at the LCR HSs were similar to TAL1 binding pattern (Figures 6B and 7B and C). Rad21 occupancy was maintained at HS5 but was increased at 3′HS1 (Figure 7D). The basis for the increase is not clear at this time but the strong binding of Rad21 might lead to the increase of cross-linking frequency between HS5 and 3′HS1 in TAL1 overexpressing cells (Figure 6E). These results suggest that increased TAL1 expression can drive increased LCR occupancy and increase the presence of the Ldb1 complex with the consequence of increased interaction frequency between the ^G^γ-globin gene and LCR and increased ^G^γ-globin gene transcription.

## DISCUSSION

**Figure 8.**
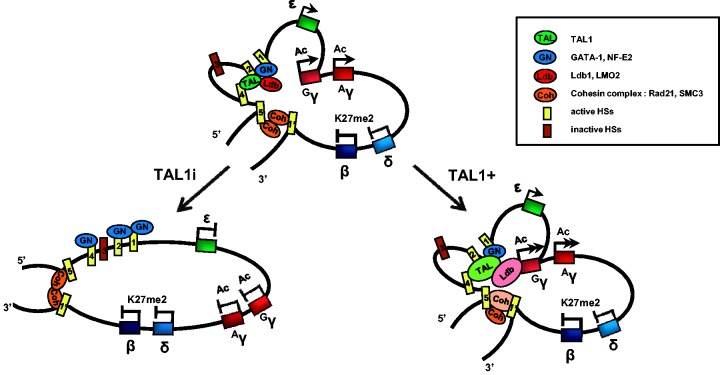
Summary of the changes by knockdown and overexpression of TAL1 in the human fetal β-globin locus in K562 cells. The results presented in Figures 1–7 were combined and represented in a model of the human β-globin locus.

This study shows that TAL1, which is one of the DNA-binding components of the chromatin loop-mediating Ldb1 complex, is required for chromatin loop formation between the LCR and ^G^γ-globin gene in human erythroid cells (Figure 8). TAL1 appears to play a role in recruiting Ldb1 and LMO2. GATA-1 occupancy in LCR is not sufficient for the recruitment of these subunits and loop formation. In addition, the data suggest that chromatin loop might be formed after several active histone modifications and HSs are established.

### Requirement for TAL1 in chromatin loop formation in the human β-globin locus

We uncovered a role for TAL1 in recruiting LMO2 and Ldb1 in the human β-globin locus, contributing to chromatin loop formation between the LCR and active ^G^γ-globin gene. The chromatin loops are disrupted by TAL knockdown and increased by its overexpression. The decrease and increase of TAL1 at the LCR HSs cause changes of LMO2 and Ldb1 binding in a parallel pattern. These results suggest TAL1 is required for recruiting these subunits in the β-globin locus. The critical role of Ldb1 in chromatin loop formation has been reported in the mouse β-globin locus; proximity between the LCR and active β-globin gene is lost on Ldb1 knockdown in MEL cells (24). A recent study using an artificial protein containing the N-terminal self-association domain of Ldb1 points to the role of Ldb1 as a bridging factor to distant sites (38). Within the Ldb1 complex, Ldb1 uses the C-terminal LIM domain to interact with the rest of the members of the complex through binding LMO2 (24). LMO2 has been reported to predominantly interact with TAL1 in hematopoietic cells, and the TAL1-LMO2 interaction has been proposed to stabilize the Ldb1 complex and modulate the activity of the complex (39).

GATA-1 is required for LCR/globin gene looping in mammalian β-globin loci, as shown in GATA-1-deficient mouse G1E cells and GATA-1 knockdown human K562 cells (6,9). Nevertheless, in our study, GATA-1 occupancy is mostly maintained at the β-globin LCR HSs in TAL1 knockdown cells and overexpressing cells, whereas chromatin loop formation in the β-globin locus is affected by loss or increase in TAL1 expression and occupancy at the LCR. These data point to the participation of TAL1, at some level, in LCR looping. The data also suggest that GATA-1 occupancy precedes TAL1 occupancy. The GATA-1 occupancy might be required for TAL1 occupancy as shown in a study using manipulable minichromosomes (24). Mutation of the GATA-1 binding site in LCR HS2 had a greater effect on reducing TAL1 occupancy than did mutation of the adjacent E box element. Taken together, the data show that GATA-1 occupancy is not enough for chromatin loop formation in the human β-globin locus and TAL1 participation is required.

### Chromatin loop formation and gene transcription

Transcription of the β-like globin genes accompanies chromatin looping with the LCR in the β-globin locus. In our TAL1 knockdown K562 cells, active histone modifications such as H3K9/14ac, H3K27ac and H3K4me2 and HSs are established across the locus, but chromatin loops are not formed and transcription of the γ-globin genes is reduced. These data indicate that these active histone modifications and HS formation are not enough for gene transcription and that the loop formation is required. When globin gene transcription is reduced by loss of transcriptional activators or co-activators such as GATA-1, EKLF, Ldb1 and Brg1 in erythroid cells, chromatin loops with the LCR are not formed (9,24,40,41). Even though loop formation by itself is not sufficient for gene transcription to a normal level in the β-globin locus (38,42–44), actively transcribed globin genes are closely positioned to the LCR, forming the loop. Therefore, chromatin looping of the gene with the LCR enhancer appears to be a necessary step for gene transcription in the β-globin locus.

Our data suggest that the active histone modifications, H3K9/14ac, H3K27ac and H3K4me2, and HS formation are likely to precede chromatin loop formation. Histones are acetylated in the β-globin locus when NF-E2 expression is reduced in K562 cells, even though HSs and a chromatin loop are not formed normally (6). HSs in the β-globin locus are formed without the loop formation as shown in this study where TAL1 was reduced. In addition, histone hypoacetylation by loss of the HATs results in failure of HS formation and loop formation in the β-globin locus (45). Although phenotypes generated by loss of a certain nuclear protein may indicate a direct role of the protein, the phenotypes might also indicate the relationship, requirement or order among the many kinds of changes in chromatin structure and organization involved in transcription activation. A reasonable scenario is that histone hyperacetylation leads to more accessible chromatin by weakening the interaction between DNA and the histone octamer, and, thus, contributes to HS formation by allowing recruitment of nucleosome remodeling complexes (46,47). The HSs might then permit the binding of transcriptional activators and loop mediating cofactors. Loop formation might then lead to the recruitment of RNA polymerase II into the gene promoter (38,48), resulting in transcription of the gene. These relationships remain to be further explored.

## SUPPLEMENTARY DATA

Supplementary Data are available at NAR Online.

## FUNDING

National Research Foundation of Korea Grant funded by the Korean Government MEST, Basic Research Promotion Fund (to A.K.) [NRF-2011-013-C00045] [NRF-2012R1A1B5001946] and the Intramural Program of the National Institute of Diabetes and Digestive and Kidney Diseases, National Institutes of Health (to A.D.) [DK075033]. Funding for open access charge: [NRF-2012R1A1B5001946].

*Conflict of interest statement*. None declared.

## Supplementary Material

Supplementary Data
