# Supplementary material for: The hematopoietic regulator TAL1 is required for chromatin looping between the β-globin LCR and human γ-globin genes to activate transcription
Source: Nucleic Acids Res. 2014 Jan 25;42(7):4283–93. doi: 10.1093/nar/gku072 (PMC3985645; doi:10.1093/nar/gku072)
Supplement: Supplementary Data [file supp_42_7_4283__index.html]

The hematopoietic regulator TAL1 is required for chromatin looping between the β-globin LCR and human γ-globin genes to activate transcription — Supplementary Data 

# The hematopoietic regulator TAL1 is required for chromatin looping between the β-globin LCR and human γ-globin genes to activate transcription

## Supplementary Data

files

**Files in this Data Supplement:**

- Supplementary Data - pdf file
