# Supplementary material for: The hematopoietic regulator TAL1 is required for chromatin looping between the β-globin LCR and human γ-globin genes to activate transcription
Source: Nucleic Acids Res. 2014 Jan 25;42(7):4283–93. doi: 10.1093/nar/gku072 (PMC3985645; doi:10.1093/nar/gku072)
Supplement: Supplementary Data [file supp_gku072_nar-01926-m-2013-File003.pdf]

Supplementary Fig. 1.

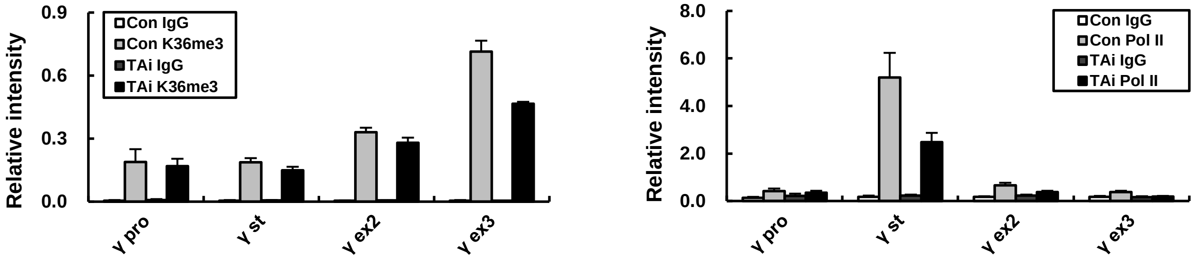

Supplementary Figure 1. Histone H3K36me3 and RNA polymerase II distribution of the γ-globin genes in TAL1 knockdown K562 cells. ChIP was performed with antibodies specific to H3K36me3 and RNA polymerase II in control and TAL1 knockdown K562 cells. Relative intensity was determined by quantitatively comparing immunoprecipitated DNA with input for the indicated amplicons and then by normalizing to the intensity of the Actin gene. Normal rabbit IgG (IgG) served as experimental control. The results of three independent experiments ± SEM are graphed.
